# Genome-Wide Identification of ARF Transcription Factor Gene Family and Their Expression Analysis in Sweet Potato

**DOI:** 10.3390/ijms22179391

**Published:** 2021-08-30

**Authors:** Isaac Seth Pratt, Baohong Zhang

**Affiliations:** Department of Biology, East Carolina University, Greenville, NC 27858, USA; prattis@ecu.edu

**Keywords:** sweet potato, auxin response factor, root development

## Abstract

Auxin response factors (ARFs) are a family of transcription factors that play an important role of auxin regulation through their binding with auxin response elements. ARF genes are represented by a large multigene family in plants; however, to our knowledge, the ARF gene family has not been well studied and characterized in sweet potatoes. In this study, a total of 25 ARF genes were identified in *Ipomea trifida*. The identified *ItrARF* genes’ conserved motifs, chromosomal locations, phylogenetic relationships, and their protein characteristics were systemically investigated using different bioinformatics tools. The expression patterns of ItfARF genes were analyzed within the storage roots and normal roots at an early stage of development. ItfARF16b and ItfARF16c were both highly expressed in the storage root, with minimal to no expression in the normal root. ItfARF6a and ItfARF10a exhibited higher expression in the normal root but not in the storage root. Subsequently, ItfARF1a, ItfARF2b, ItfARF3a, ItfARF6b, ItfARF8a, ItfARF8b, and ItfARF10b were expressed in both root types with moderate to high expression for each. All ten of these ARF genes and their prominent expression signify their importance within the development of each respective root type. This study provides comprehensive information regarding the ARF family in sweet potatoes, which will be useful for future research to discover further functional verification of these ItfARF genes.

## 1. Introduction

Indole-3-acetic acid (IAA), one type of auxin, is a plant growth hormone that elicits developmental growth throughout the plant’s life cycle, including seed germination, vascular tissue formation, and reproductive and vegetative growth [1,2]. These auxin plant hormones work in conjunction with auxin response factors (ARF), which are a set of transcription factors. When ARFs are bound to auxin response elements (AuxRE, TGTCNN), they work to activate or repress auxin gene expression and regulation [3,4,5]. General genome-wide AuxRE is TGTCNN and in particular TGTCGG for ARF5 but not TGTCTC [5]. ARF proteins can be broken down into three domain subunits: an amino-terminal DNA-binding domain (DBD), a conserved carboxy-terminal dimerization domain (CTD), and a non-conserved middle domain (MD) [2]. The DBD for ARF’s are found in the N-terminal region and bind specifically to the AuxRE TGTCNN in promoters for the regulation of auxin gene expression [6]. Carboxy-terminal dimerization domains facilitate both homo- and hetero-dimerization through protein interactions, relating themselves to the domain III and IV in Aux/IAA proteins [6]. The MD has a double function, acting as either a repression domain (RD) or an activation domain (AD) [7,8]. ARF proteins that have a MD rich in glutamine (Q) are constituted as an activator; this can be seen in AtARF5, 6, 7, 8, and 19 [9,10], while ARF proteins that have an MD rich in Proline (P), Serine (S), and Threonine (T) are considered repressors; these repressors were found in AtARF1, 2, 3, 4 and 9 [8]. ARF proteins have been identified in a variety of plants; 22 ARF genes and one pseudogene were found in *Arabidopsis* [7], and 25 ARF genes were found in rice [11]. Subsequently, there have been more ARFs identified in a multitude of other plant species, such as tomato (*Solanum lycopersicum*) [12], kiwifruit (*Actinidia chinensis*) [13], longan (*Dimocarpus longan* L.) [14], tomato (*Solanum lycopersicum*) [15], sorghum (*Sorghum bicolor*) [16,17], soybean (*Glycine max*) [18], maize (*Zea maize)* [19], Chinese cabbage (*Brassica rapa*) [20], cotton (*Gossypium raimondii*) [21], sweet orange (*Citrus sinensis*) [22], peach (*Prunus persica L.*) [23], and alfalfa (*Medicago truncatula)* [24]. However, with all these identifications of auxin response factors, there has not been the identification of auxin response factors in sweet potato.

The sweet potato (*Ipomoea batatas*) of the family Convolvulaceae is a highly desired food crop throughout the world and has been ranked the 7th largest crop. It serves as a staple diet in developing countries for its overall high nutritional and caloric values, ability to grow in most climates and conditions, and having a high production yield. The sweet potato (*Ipomoea batatas*) is a hexaploid with 90 chromosomes, making genomic research on the crop highly complicated [25]. However, there is an alternative to performing genomic research on sweet potatoes, and that is to use a diploid relative. The diploid *Ipomoea trifida* is a relative to the hexaploid *I. batatas* and is a model species of genomic research due to its small genome size and number of chromosomes [26,27,28]. In this study, all the potential ARF transcription factors from the *I. trifida* genome were identified using various bioinformatics tools. ItfARF proteins, once identified, underwent a phylogenetic analysis where they were compared among themselves and then amongst the model plant species: *Arabidopsis, S. lycopersicum, O. sativa,* and *G. raimondii.* Finally, the expression profiles of each ItfARF gene were analyzed using tissue samples of both the normal and storage roots of sweet potato plant *I. trifida*.

## 2. Materials and Methods

### 2.1. Identification of ARF Genes in I. trifida

The genome sequence of *Ipomoea trifida* was downloaded from the Sweet potato genomic resource database (http://sweetpotato.plantbiology.msu.edu/, accessed on 1 June 2021) [29]. Both a BLAST and reciprocal BLAST search were performed for the conformation of *I. trifida* ARF gene families, and orthologues relations with the species: *Arabidopsis, S. lycopersicum, G. raimondii,* and *O. sativa.* For the identification of the genomic members of the ARF gene family, both HMMER 3.3.1 (http://hmmer.org/download.html, accessed on 1 June 2021) and Pfam 33.1 (http://pfam.xfam.org/, accessed on 1 June 2021) were used to search whole genome sequences with Pfam’s ARF (PF06507); all sequences with an e-value < e-10 were selected after the homology search. All obtained sequences were further analyzed for B3, ARF, and AUX/IAA domains using NCBI’s CDD (https://www.ncbi.nlm.nih.gov/cdd/, accessed on 1 June 2021). After identification of the ItfARF gene family, information on the ItfARF’s genome location, protein, and CDS length were obtained along with the gene, protein, and CDS sequences from the Sweet potato database. In addition, Expasy (https://web.expasy.org/compute_pi/, accessed on 1 June 2021) was used to compute the isoelectric point (PI) and the molecular weight (Mw) of the ItfARF proteins. To analyze the identified ItfARF genes for conserved motifs, the protein sequences were examined using the software MEME (Multiple Expectation maximizations for Motif Elicitation) (https://meme-suite.org/meme/tools/meme, accessed on 1 June 2021). The sequence search options were as follows: motif distribution among sequences was zero to one occurrence, the motif width range was from 6 to 50 amino acids, and the maximum motifs per sequences was 20.

### 2.2. Phylogenetic Analysis of ARF Genes

To better understand the evolutionary relationship and homology between the sequences, a phylogenetic analysis was performed using the protein sequence data [30]. For this analysis, the protein sequences from *Arabidopsis, G. raimondii, I. trifida, O. sativa*, and *S. lycopersicum* were analyzed with Molecular Evolutionary Genetics Analysis (MEGA-X 10.2) using the maximum-likelihood method and its built-in sequence alignment tool, Clustal-W [30].

### 2.3. Sweet Potato Culture, Tissue Collection, and RNA Extraction

The sweet potatoes (*Ipomoea batatas*) were cultivated in the greenhouse with normal agronomic practices. Both storage root and normal root samples were collected from 5-weeks grown sweet potato plants. Root tissues were quickly sampled from the plants, immediately frozen in liquid nitrogen, and stored at −80 °C until RNA extraction. At least six biological replicates were collected for each type of root. Total RNAs were extracted from each root sample by using a mirVana^TM^ miRNA isolation Kit (Ambion, Austin, TX, USA) as performed in our previous studies [31,32,33,34]. Briefly, the tissues were ground into a fine powder in a mortar and pestle and then transferred into a 2 mL centrifuge tube with the Lysis/Binding buffer. For the normal roots, 400 μL of the Lysis/Binding buffer was used, and for the storage roots, 700 μL buffer was used; this is because the storage root contained higher levels of starch that absorbed large quantities of liquid. Then, the samples were sonicated for 15–20 s on ice. Post sonication, each sample was inverted for an accurate mixture of the Lysis buffer and the plant material. After a 10-min ice bath, 400 μL of Acid-Phenol/Chloroform was added for the separation of RNA from its cellular components. Carefully following the manufacturer’s protocols, consisting of aqueous phase extraction and several wash cycles, 95 ℃ nuclease-free water was added to the filter cartridge medium for the conclusion of RNA isolation. To test the concentration and purity of the RNA isolated, Nanodrop ND-1000 was used. Samples NR1, NR2, NR3, SR1, SR2, and SR3 were all collected, each having a ng/μL concentration in the range of 200–350 ng/μL.

### 2.4. qRT-PCR

The RNA of each plant tissues sample was prepared for reverse transcription with specific primers designed from NCBI primer design tool. Then, reverse transcription was performed by following the instructions of the TaqMan^®^ MicroRNA Reverse Transcription Kit (Applied Biosystems, CA, USA). This kit included a Multiscribe^TM^ Reverse transcriptase (50 U/μL), dNTPs with dTTP (100 nm), Reverse transcription buffer (10x), RNase inhibitor (20 μ/μL), nuclease-free water, and total RNAs; the volume for each reaction was 15 μL. Post reaction completion, 150 μL of nuclease-free water was added to the products and stored at −20 °C until ready for qRT-PCR. qRT-PCR was performed on a 96-well plate within the 7300 Fast Real-Time PCR System (Applied Biosystems, Waltham, CA, USA). The samples ran for each replicate went as follows: all ItfARF genes and 2 reference genes (EF1α and UBC). SYBR Green was used to analyze gene expression within the qRT-PCR system. A total of 6 reactions were run: three biological replicates for each root type, and each biological replicate had three technical replicates. The reaction temperature program settings were as follows: 10 min at 95 °C, with 40 cycles of 15 s at 95 °C, and 60 s at 60 °C.

### 2.5. Data and Statistical Analysis

Statistical analysis was performed after obtaining sample triplicate Ct values. Elongation factor 1α (EF1α) and ubiquitin-c (UBC) served as the reference genes, and the average of each reference gene’s ∆Ct values was combined and subtracted from all other ItfARF genes ∆Ct values to obtain a second normalization. Differentially expressed genes were discovered using statistical calculation, *p* < 0.05. Then, fold change was calculated for each gene using the formula: 2^−(∆∆Ct)^. A hierarchical clustering analysis was performed using Multi Experiment Viewer (MeV) to create a heat map of gene expression for all the ItfARF genes.

## 3. Results

### 3.1. Identification and Sequence Analysis of ARF Genes in I. trifida

**Figure 1:**
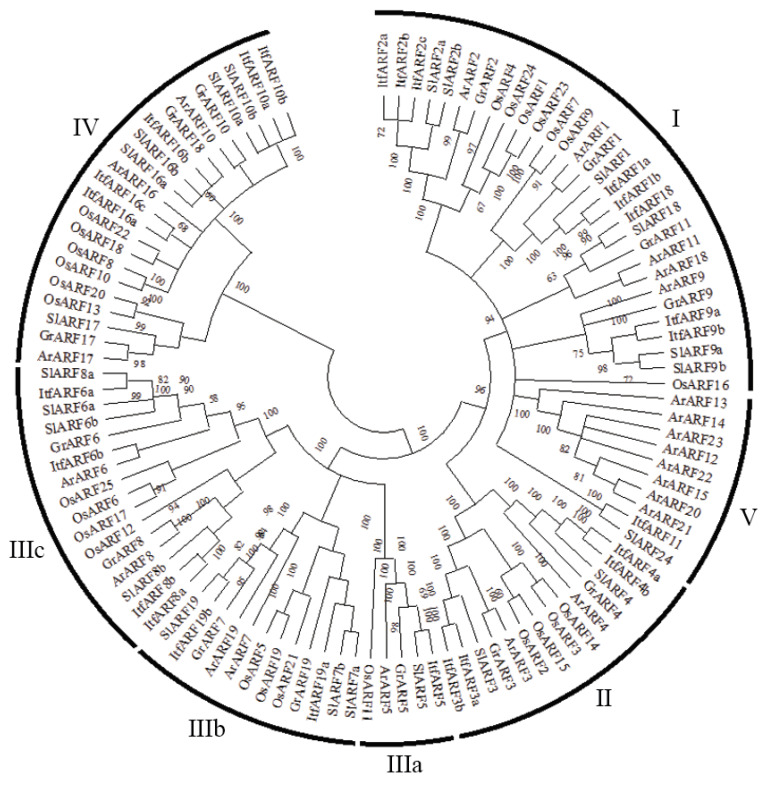
Phylogenetic tree comprised of ARF proteins found in *Arabidopsis*, cotton, *I. trifida*, tomato, and rice. This tree was constructed using MEGA-X software using the maximum-likelihood method with the JTT model. The parameters were 1000 bootstraps and pairwise gap deletions. The 109 ARF proteins were classified into five classes: **I**, **II**, **III**, **IV**, and **V**, and class III was further divided into **IIIa**, **IIIb**, and **IIIc**.

To identify the ARF transcription factor genes in *I. trifida*, the ARF protein domain (PF00025) was used to blast search against the *I. trifida* genome. The first search led to a total of 282 possible protein sequences in *I. trifida*. After eliminating the redundant sequences and comparing with the ARF gene in other plant species (Figure 1), a total of 25 ItfARF were identified in the sweet potato genome (Supplementary Materials Table S1). Then, these 25 sequences were compared and categorized into 13 different ARF gene subfamilies, including ItfARF1, ItfARF2, ItfARF3, ItfARF4, ItfARF5, ItfARF6, ItfARF8, ItfARF9, ItfARF10 ItfARF11, ItfARF16, ItfARF18, and ItfARF19 named so after their similarities to *Arabidopsis*. The average number of exons in the gene sequences was 10, having a similar distribution to the *Arabidopsis* ARF genes. The length of the CDS varied from 1731 bp (ItfARF11) all the way up to 3375 bp (ItfARF19a). The 25 possible genes all produced adequate proteins ranging from 576 to 1124 amino acids in length. The predicted MW of the ItfARF were as low as 64.539 kDa and as high as 124.164 kDa. The PI values of the ItfARF genes were from 4.99 to 9.44; most of these values fell in the range of 5–7, suggesting they encode weak acid proteins, while those few that ranged from 7.56 to 9.44 encode for weak basic proteins.

**Figure 2:**
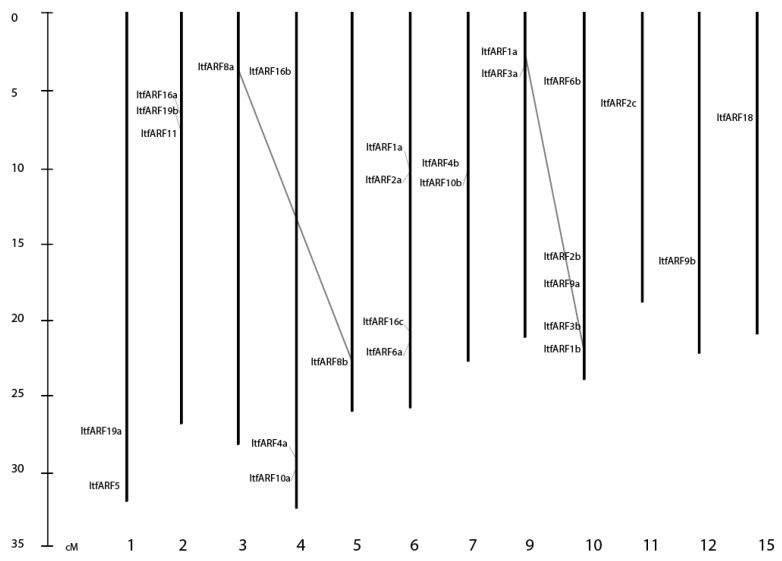
Chromosomal distribution of ARF genes in *I. trifida*, spanning 12 of the 15 chromosomes, gene duplication analysis of ItfARF was presented with a gray line.

The 25 identified ARF genes were located among the 15 chromosomal pairs with the exclusion of Chr. 8, 13, and 14 as no ARF genes were mapped there. Most of the genes stacked onto a select few chromosomes (Figure 2), having five genes on chromosome 10 (19.2%), four genes on chromosome 6, three genes each on chromosomes 2, and 4, two genes each on chromosomes 1, 7, 9, and 11, and then one gene on chromosomes 3, 5, 12, and 15. The ARF genes, ItfARF8a and ItfARF8b and ItfARF1a and ItfARF1b, are the only two sets of duplicate genes that met the 80% sequence similarity for each of their respective nucleotide sequences.

### 3.2. Conserved Domains and Motif Analysis of ItfARF Proteins

**Figure 3:**
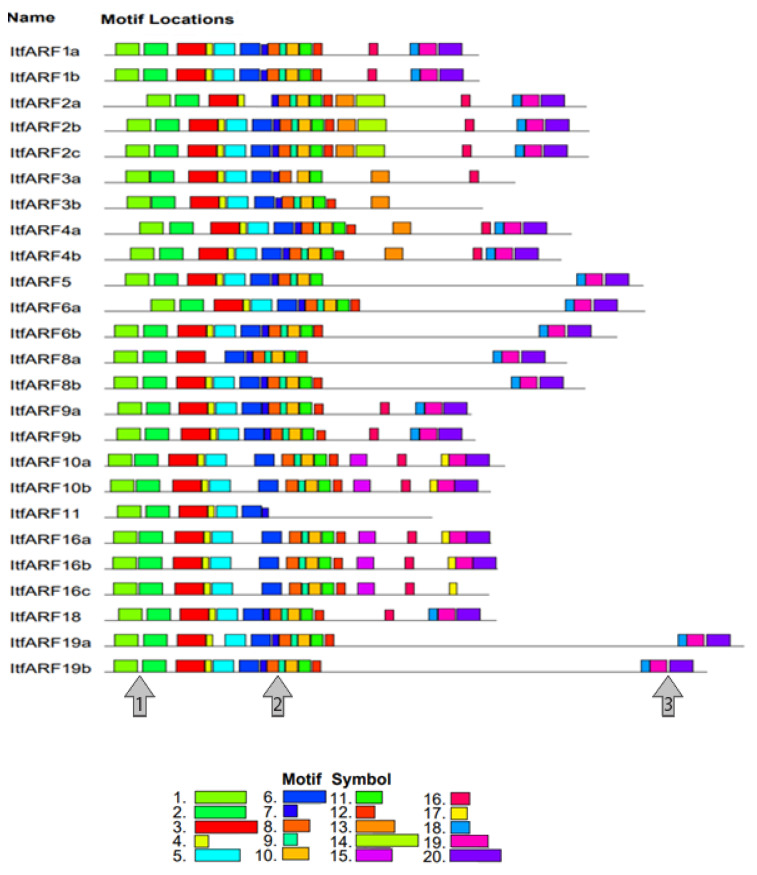
ItfARF proteins with twenty different identified conserved motifs through the MEME search tool. The motifs are numbered and represented by the different colors in Motif Symbol. Arrow 1 points to the DBD domain, Arrow 2 points to the ARF domain, and Arrow 3 points to the CTD domain.

For the prediction of protein function, the analysis of both domains and subdomains was apparent [2]. Out of the twenty-five ItfARF proteins, there were four among them that lacked at least one of the three typical domains, and each of these four genes were lacking the Aux/IAA CTD domain; those four genes were ItfARF3a, ItfARF3b, ItfARF11, and ITFARF16c (Supplementary Materials Table S1). Regarding the domains, the middle domain (MD) gave insight into the sequences’ ability to either be a transcriptional activator or a repressor. The protein sequences that were rich in (Q) have been regarded as activators ItfARF5, 6a, 6b, 8a, 8b, 19a, and 19b, while the other protein sequences whose MD were rich in P, S, and T are regarded as transcriptional repressors. The conserved motif analysis allowed for further confirmation of these notions. As shown in Figure 3, motifs 1 and 2 correspond to a DNA-binding domain; motifs 8, 9, 10, and 11 correspond to an ARF domain, while motifs 17, 18, 19, and 20 correspond to an Aux/IAA domain. Lastly, all seven ItfARF transcriptional activators lack motif 16 while still maintaining motifs 18, 19, and 20, suggesting that this combination of motifs signifies a transcriptional activator. Cross-comparing Supplementary Materials Table S1 and Figure 3, we can see that these motifs correlate well with each other. Each protein sequence had a variable number of motifs, but the motifs for each domain were conserved with minimal variation preceding and succeeding them.

### 3.3. Phylogenetic Analysis of ItfARFs

For better understanding the functional and evolutionary relationship of the ARF gene family, an unrooted phylogenetic tree was created by multiple sequence alignment in MEGA-X for all selected plant species, including sweet potato. This analysis used the 25 ItfARF protein sequences and all the ARF proteins from *Arabidopsis,* rice, cotton, and tomato. The 109 ARF transcription factors from all species fall into five major classifications ranging from I to IV; the classes were derived from their phylogenetic relationship. Out of all the classes, Class III was the largest, having 33 ARF members holding 30.27% of the total ARF genes analyzed. Class III was comprised of three subgroups: IIIa, IIIb, and IIIc; these contained 5, 12, and 16 ARF genes, respectively. Classes IIIa, IIIb, and IIIc contained ItfARFs with a Q-rich MD, which included ItfARF5 for IIIa, ItfARF6a, 6b, 8a, and 8b for IIIc, and 19a and 19b for IIIb. ItfARFs were in each class except for class V, suggesting that the ItfARF gene family arose before the lineage spilt.

### 3.4. Expression Profiling of ItfARF Genes in Root Tissues

**Figure 4:**
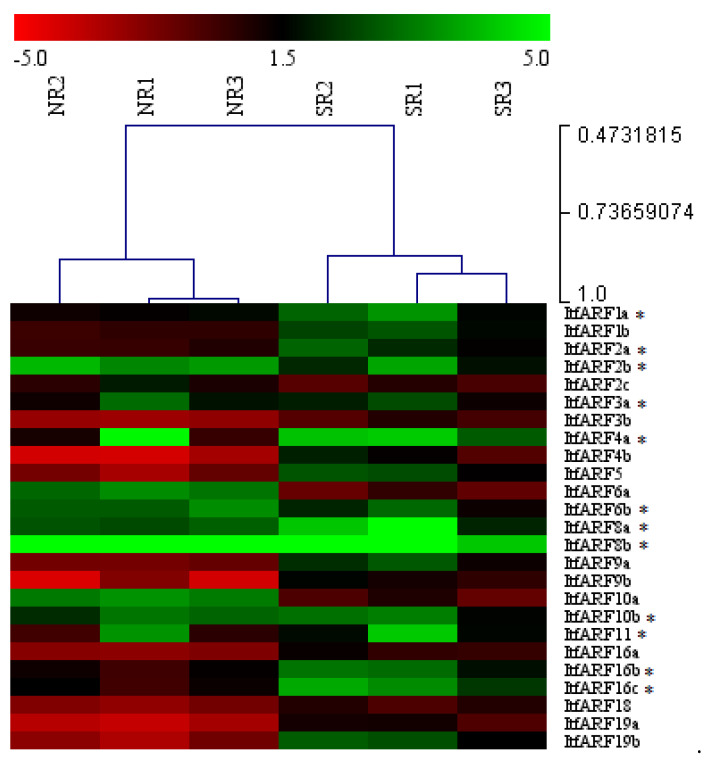
Heat map of ARF gene expression within all six biological samples of both the normal root and the storage root. The tissue samples are named from left to right at the top of the figure, and the names of the 25 genes are directly to the right of the figure. The expression rankings are shown using a range of color: higher gene expression is represented by green; lower expression, red; and median expression, black. *’s denotes which genes are statistically different, * *p* < 0.05.

qRT-PCR was employed to better understand the expression profiles of all ItfARF genes within sweet potato normal roots and storage roots. Through the gene expression analysis, the differing functions of all ItfARF genes were apparent. There were some ItfARF genes with similar gene expression patterns between the two root types, while other genes showed clear differences, with higher expression in one root type over the other (Figure 4). For example, ItfARF8a and ItfARF8b were both highly expressed in both normal roots and storage roots. ItfARF2b, ItfARF3a, ItfARF6b, and ItfARF10b are all moderately expressed across both tissue types, suggesting that they may be primarly expressed under specific conditions. Only two of the 25 tested ItfARF genes were more highly expressed in the normal root than that in the storage root; those genes were: ItfARF6a and ItfARF10a. There were some genes that had little to no expression at all, such as ItfARF2c, ItfARF3b, ItfARF9b, ItfARF16a, ItfARF18, and ItfARF19a; these suggest that the rest of the ItfARF genes were expressed more highly in the storage roots as opposed to the normal roots, those genes being ItfARF1a, ItfARF1b, ItfARF2a, ItfARF4a, ItfARF4b, ItfARF5, ItfARF9a, ItfARF16b, ItfARF16c, and ItfARF19b. Out of those nine genes, ItfARF4a, ItfARF5, ItfARF9a, ItfARF16b, ItfARF16c, and ItfARF19b had the highest level of expression in the storage roots.

## 4. Discussion

### 4.1. ARF Genes in I. trifida

ARF are responsible for the regulation of multiple plant processes, including lateral root formation, fruit initiation, apical dominance, and cellular senescence [35]. ARF genes have been continuously reported throughout many different plant species, but they have not been reported within sweet potatoes, an emerging biofuel crop. In the present study, 25 ARF transcription factor genes belonging to 13 ARF gene families were identified and characterized in *I. trifida*. Overall, the number of ItfARF genes surpassed those in other plant species, such as *Arabidopsis* rice and tomato; however, when categorized into gene families, *I. trifida* had the smallest amount of ARF gene family representation at 13 [2]. This gives rise to the question: what happened in the evolutionary process between *Arabidopsis*, rice, tomato, and *I. trifida* that caused a disparity in total gene number? The gene family presence might have been decreased throughout the evolution of the species. This can be seen in Figure 1, where there are no ItfARF, OsARF, SlARF, or GrARF genes in classification V, possibly meaning that those AtARF genes were derived from a singular AtARF gene [21]. There is also the notion that *I. trifida* has gone through genomic rearrangements due to gene duplication, giving rise to a higher quantity of ItfARF genes. Genes can also lose function due to genomic rearrangements, such as ItfARF3a, as it does not contain a CTD domain. An important note on the reduction of gene family representation in *I. trifida* regards the nomenclature system in place. All the ItfARF genes were named according to the most accurate sequence similarity of ARF proteins in *Arabidopsis*.

### 4.2. Phylogenetic Characterization and Function of ARF Gene Family

The ARF transcription factor orthologous relationships can be seen throughout the phylogenetic analysis. Those relationships between *Arabidopsis* and *I. trifida* are almost constant for each ItfARF gene, save for ItfARF11. This implies that the orthologous classes I–IV are conserved, but it does not imply that the function of the ARF genes in *I. trifida* are directly related to the *Arabidopsis* ARF gene functions. The ARF genes across species may be functionally different, either because of plant specific evolutions or because of gene duplication. Regarding previous studies, ARF double-mutant genes typically have a more pronounced phenotype than their single-mutant counterpart, implying that closely related ItfARF genes may be functionally redundant for plant growth [36,37]. As seen in *Arabidopsis*, ARF2 regulatory functions played multiple roles relating to plant aging such as flower initiation, rosette leaf senescence, and floral organ abscissions. Then, ARF1 mutations were seen to enhance those ARF2 regulatory phenotypes, suggesting that ARF1 is partially redundant with ARF2 [38]. ARF7 and ARF19 mutations had functional redundancies between both genes, which impacted and impaired lateral root formation and abnormal gravitropism in both the hypocotyl and the root [36,39,40]. ARF4 double mutants enhance ARF3 mutants regarding ARF3′s role in gynoecium abaxial identity [41]. ARF6 and ARF8 both regulate stamen and gynoecium maturation. In single mutations of ARF6 and ARF8, flower maturation was delayed, reducing fertility, while the double mutants were completely infertile [42]. ARF8 has also been seen to affect hypocotyl elongation and root growth habit regarding light sensitivity, indicating a possible dominant role in the development of *I. trifida* [43]. ARF10 and ARF16 double mutants display abnormal root cap gravitropism and root cap developmental defects [44]. Each of these mutants in the phylogenetic tree all fall together under their classes: ARF1 and ARF2, ARF3 and ARF4, ARF6 and ARF8, and ARF10 and ARF16 are all in classes I, II, III, and IV respectively. This mutation classification relationship suggests that functionally redundant genes have close evolutionary relationships.

For the ItfARF chromosomal distribution, it should be noted that no ARF genes were located on chromosomes, 8, 13, or 14, whereas in *O. sativa*, there is an ARF gene on chromosome 8, suggesting that the ARF gene might have been lost or transferred to a different chromosome during the species evolutionary history.

### 4.3. Gene Expression of ItfARF Genes in Root Tissues

**Figure 5:**
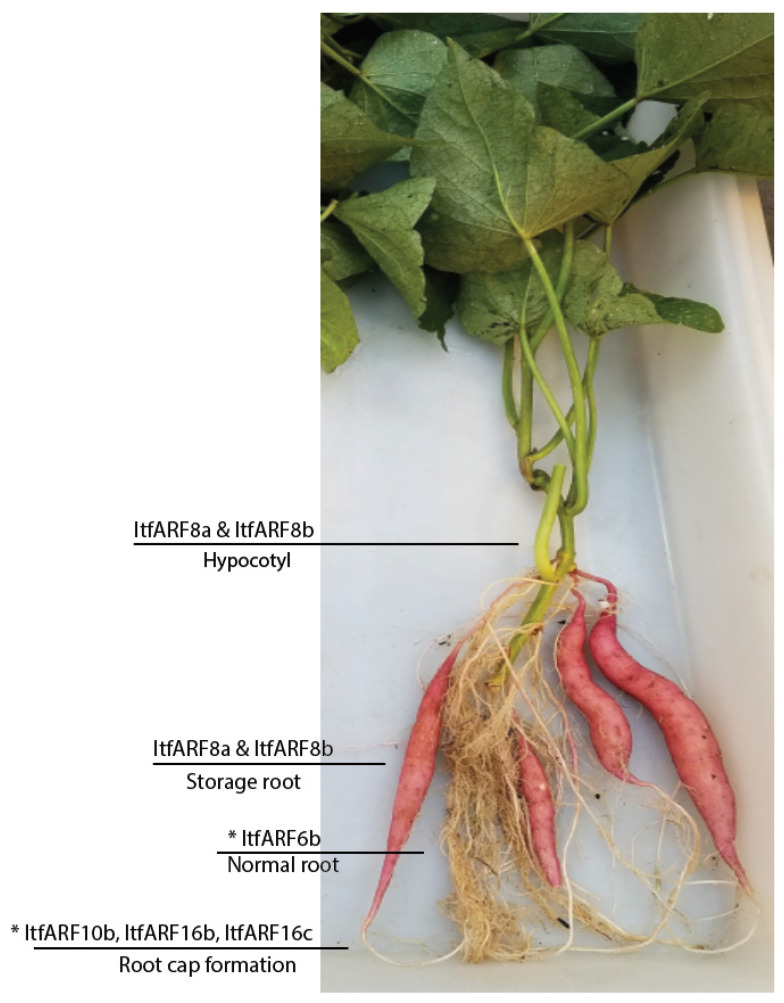
Five-week-old *I. batatas* normal root and storage root samples. Areas labeled correspond to where those genes are most likely expressed. An * denotes ItfARF8a and ItfARF8b, as their function is predicted to affect overall root growth and development.

Protein data such as motifs and domains are useful for the prediction of novel gene function. Through sequence analysis of all the ItfARF protein sequences, it was found that only seven of the 25 genes contained a middle region rich in Q, meaning these seven genes most likely act as activators: those genes being ItfARF5, ItfARF6a, ItfARF6b, ItfARF8a, ItfARF8b, ItfARF19a, and ItfARF19b. The remaining 18 genes rich in SPT and not in Q are most likely acting as transcriptional repressors. In *Arabidopsis*, the ARF genes that act as activators are ARF5, ARF6, ARF7, ARF8, and ARF19. All the activators for both *Arabidopsis* and *I. trifida* fall into the same phylogenetic classification of class III (Figure 1), suggesting that the activation genes and domains are conserved through the evolutionary process. The study also showed the root-specific expression profiles of ARF transcription factors in sweet potato. The results showed that the majority of ARF genes were more active in the storage root, with only nine genes in total that had moderate to high expression levels within the normal roots. Out of those nine ARF genes, there were four ItfARF activators, those being ItfARF6a, ItfARF6b, ItfARF8a, and ItfARF8b. Of those four ARF genes, ItfARF6a was the only transcriptional activator that was solely expressed in the normal root; the other three were expressed equally across both root types; this suggests that ItfARF6a is a main activator of the normal root growth. ItfARF8a and ItfARF8b were highly expressed across both root tissue types, suggesting that these two ItfARF genes are the most prevalent activators for the entire sweet potato root system. As for the other three ItfARF gene activators, ItfARF5 and ItfARF19b both have moderately high expression levels in the storage root alone, while ItfARF19a has almost no expression at all, meaning ItfARF19a might be expressed more in the hypocotyl than the entire root itself, and ItfARF5 and ItfARF19b are activators for storage root development. Relating expression levels to evolutionary history with other species, both ItfARF10 and ItfARF16 gene sets have relative expression regarding the root system of *I. trifida.* ItfARF10a is almost exclusively expressed in the normal roots, ItfARF10b is shown to have moderate expression across both roots, and ItfARF16b and ItfARF16c are expressed in both roots; however, the storage root expression levels are much higher. This relates back to the ARF10 and ARF16 genes in *Arabidopsis* that affect root cap gravitropism and root cap development [44]. Furthermore, all ItfARF10 genes and ItfARF16 genes were classified together in class IV of both phylogenetic trees, meaning not only are these genes functionally similar, but they are also evolutionarily similar. These results suggest that ItfARF genes play important roles in both normal and storage root development (Figure 5). Further research should be conducted to investigate the ItfARF genes for further functional verification of their role in sweet potato root systems, namely those seven ItfARF genes that specifically expressed in the storage root by more advanced biotechnological tools such as transgenics and CRISPR/Cas9 gene editing [45,46].

## Data Availability

All data are reported in this manuscript.

## Supplementary Materials

The following are available online at https://www.mdpi.com/article/10.3390/ijms22179391/s1, Table S1: The characteristics of identified ARF gene families in *I. trifida*.
