# Supplementary material for: Genome-Wide Identification of ARF Transcription Factor Gene Family and Their Expression Analysis in Sweet Potato
Source: Int J Mol Sci. 2021 Aug 30;22(17):9391. doi: 10.3390/ijms22179391 (PMC8431151; doi:10.3390/ijms22179391)
Supplement: Supplementary file 1 [file ijms-22-09391-s001.zip › ijms-1302712-SI.pdf]

Table S1. The characteristics of identified ARF gene families in *I. trifida*

Gene Name	Gene Symbol	CDS length (bp)	Domains	Deduced protein			Chr	Genome location	Exon Number
				Length (aa)	MW (kDA)	PI			
ItfARF1a	itf09g04490	1977	B3, ARF, AUX/IAA	658	73.862	5.86	9	2229243 - 2223211	14
ItfARF1b	itf10g21760	1980	B3, ARF, AUX/IAA	659	74.102	5.97	10	22768345 - 22773913	14
ItfARF2a	itf06g08230	2652	B3, ARF, AUX/IAA	884	98.724	6.72	6	10610618 - 10605949	7
ItfARF2b	itf10g13730	2559	B3, ARF, AUX/IAA	852	94.263	6.38	10	16445652 - 16440359	14
ItfARF2c	itf11g10870	2556	B3, ARF, AUX/IAA	851	94.731	6.09	11	6559617 - 6564877	14
ItfARF3a	itf09g07030	2169	B3, ARF	722	79.299	6.57	9	3677006 - 3671593	10
ItfARF3b	itf10g19820	1998	B3, ARF	665	73.034	7.72	10	21532579 - 21536723	10
ItfARF4a	itf04g30540	2466	B3, ARF, AUX/IAA	821	90.894	6.75	4	29721501 - 29715473	12
ItfARF4b	itf07g11450	2412	B3, ARF, AUX/IAA	803	89.081	5.56	7	9997889 - 9991527	12
ItfARF5	itf01g35200	2844	B3, ARF, AUX/IAA	947	105.019	5.2	1	31640623 - 31635815	13
ItfARF6a	itf06g20390	2853	B3, ARF, AUX/IAA	950	105.591	6.33	6	22207636 - 22201198	14
ItfARF6b	itf10g04850	2706	B3, ARF, AUX/IAA	901	99.541	6.01	10	4527118 - 4520349	14
ItfARF8a	itf03g05320	2442	B3, ARF, AUX/IAA	813	90.204	5.62	3	3300707 - 3306419	14
ItfARF8b	itf05g23120	2538	B3, ARF, AUX/IAA	845	93.964	5.82	5	23445845 - 23452495	14
ItfARF9a	itf10g16260	1938	B3, ARF, AUX/IAA	645	72.391	6.69	10	18600817 - 18605381	14
ItfARF9b	itf12g17310	1959	B3, ARF, AUX/IAA	652	73.094	6.31	12	16906880 - 16902499	14
ItfARF10a	itf04g30820	2115	B3, ARF, AUX/IAA	704	77.748	7.83	4	29891980 - 29887654	4
ItfARF10b	itf07g11640	2040	B3, ARF, AUX/IAA	679	74.738	7.56	7	10271997 - 10268425	4
ItfARF11	itf02g08750	1731	B3, ARF	576	64.539	4.99	2	8156302 - 8152920	11
ItfARF16a	itf02g08580	2043	B3, ARF, AUX/IAA	680	75.246	6.53	2	8048900 - 8051867	3
ItfARF16b	itf04g06590	2076	B3, ARF, AUX/IAA	691	75.873	6.56	4	3914771 - 3910977	3
ItfARF16c	itf06g19540	2031	B3, ARF	676	74.815	5.75	6	21612765 - 21615756	2
ItfARF18	itf15g10150	2070	B3, ARF, AUX/IAA	689	76.415	6.22	15	6848850 - 6843635	14
ItfARF19a	itf01g29370	3375	B3, ARF, AUX/IAA	1124	124.164	6.62	1	27916897 - 27923035	14
ItfARF19b	itf02g07020	3180	B3, ARF, AUX/IAA	1059	117.923	6.05	2	7111545 - 7105401	13
